# Gestational Diabetes Mellitus Does Not Worsen Obstetrical and Neonatal Outcomes of Twin Pregnancy

**DOI:** 10.3390/jcm12093129

**Published:** 2023-04-26

**Authors:** Alice Ronco, Sofia Roero, Silvana Arduino, Arianna Arese, Isabella Ferrando, Gabriella Scaltrito, Viola Casula, Teresa Fea, Mattia Mazza, Carlotta Bossotti, Roberto Zizzo, Alberto Revelli

**Affiliations:** Gynecology and Obstetrics 2U, Sant’Anna Obstetric Gynecological Hospital, A.O.U. Città della Salute e della Scienza, Corso Spezia 60, 10126 Torino, Italy

**Keywords:** twin pregnancy, gestational diabetes mellitus, pregnancy outcome

## Abstract

The specific effects of gestational diabetes mellitus (GDM) on twin pregnancy outcomes, which are at high risk per se, are unclear. The present study analyzes outcomes of twin pregnancies complicated by GDM (n = 227) by comparing them with GDM singleton pregnancies (n = 1060) and with twin pregnancies without GDM (n = 1008), all followed up at Sant’Anna Hospital, Turin (Italy), between January 2010 and March 2020. The prevalence of GDM among twin pregnancies (n = 1235) was 18.4%. Compared to GDM singletons, GDM twins had higher rates of preeclampsia (aOR 2.0; 95% CI 1.2–3.8), cesarean section (aOR 7.5; 95% CI 5.2–10.8), and neonatal hypoglycemia (aOR 2.5; 95% CI 1.1–5.3). They had a higher incidence of abnormal 2 h OGTT values (aOR 7.1; 95% CI: 3.2–15.7) and were less likely to require insulin therapy (aOR 0.5; 95% CI: 0.3–0.7). In comparison with twin pregnancies without GDM, women with GDM twins were significantly older (35.0 vs. 33.0 years; *p* < 0.001) and had higher BMI (23.0 versus 22.0 kg/m^2^; *p* < 0.001); they had a higher incidence of LGA newborns (aOR 5.3; 95% CI 1.7–14.8), and lower incidence of low APGAR scores (0.5; 95% CI 0.3–0.9). Overall, GDM does not worsen outcomes of twin pregnancy, which is per se at high risk for adverse outcomes.

## 1. Introduction

Gestational diabetes mellitus (GDM) is a common complication of pregnancy; its incidence, generally around 6%, is gradually rising due to the increasing prevalence of advanced maternal age and obesity, two well-known risk factors [1,2]. Additionally, the incidence of twin pregnancy has progressively grown in the last decades, as a result of both the rise in maternal age and the widespread use of infertility treatments; overall, it is now reported at 2–4% of all pregnancies [3]. Recent evidence suggests that twin pregnancy is an independent risk factor for GDM, probably due to the increased placental mass, which results in higher levels of placental lactogen [4,5,6,7]. 

In singleton pregnancy, GDM is associated with adverse obstetric, fetal, and neonatal outcomes, such as preeclampsia, preterm delivery, fetal macrosomia, shoulder dystocia, the need for cesarean section, and neonatal hypoglycemia [1,8]. GDM also seems to be associated with congenital malformation, although to a lesser extent than overt diabetes [9,10,11,12]. Rather surprisingly, data about the effects of GDM on the outcome of a twin pregnancy are quite scarce and conflicting. 

In twin pregnancies complicated by GDM, a higher incidence of hypertensive disorders [13,14,15], cesarean delivery [4], NICU admission [13], respiratory distress syndrome (RDS) [16], and neonatal hypoglycemia [17] was reported; some of these observations, however, were not confirmed by other studies [18,19,20]. In addition, only a few published studies compared the effects of GDM in twin pregnancy vs. GDM in singleton pregnancy [4,13,17,21,22,23,24].

Some contradictory evidence may arise from the fact that twin pregnancy is per se at risk for adverse outcomes [25,26], and complications such as preeclampsia and neonatal hypoglycemia are common in twin pregnancy irrespective of the presence of GDM [4,26]. On the other hand, some of the typical complications of GDM, such as macrosomia and shoulder dystocia, are unlikely to occur in twin pregnancies, as delivery is often anticipated at 36–37 weeks. It should also be noted that the presence of two fetuses greatly increases glucose consumption and maternal basal metabolic rate [27], so a certain degree of hyperglycemic state may be considered as part of the physiologic adaptation in twin pregnancy [28]. In fact, an association between enhanced glycemic control and increased incidence of small for gestational age (SGA) twin newborns, along with no improvement of other outcomes, was reported [29]; coherently, the attenuation of some adverse outcomes was actually seen in twin pregnancies complicated by GDM compared to those without GDM, such as, indeed, frequency of small for gestational age (SGA) newborns and low 5 min APGAR scores [15,30]. Thus, it remains unclear whether strict glycemic control is beneficial in twin pregnancy with GDM, and what should be the optimal glycemic target.

In order to contribute to the knowledge in this area, we designed the present study with the aim to evaluate maternal and perinatal outcomes of twin pregnancies complicated by GDM vs. (a) those of singleton pregnancies with GDM, and (b) those of twin pregnancies not complicated by GDM. 

Our research suggests that GDM does not worsen obstetric and neonatal outcomes of twin pregnancy, which is per se at high risk of adverse outcomes. Our data regarding glucose plasma levels may imply that there are significant differences in the physiology of glucose metabolism between twin pregnancies and singletons: this should be considered when assessing glucose tolerance and optimal plasma glucose levels in twin pregnancies.

## 2. Materials and Methods

We performed a retrospective cohort study involving twin pregnancies followed by the Twin Pregnancy Care Unit (TPCU) of Sant’Anna Hospital, Turin (Italy), who had their first visit starting 1 January 2010 until 31 March 2020. Data about singleton pregnancies with GDM delivering in our hospital in the same period were also collected. Because of anonymous data collection, the study was exempt from approval by the local institutional review board. The study fully adhered to the World Medical Association Declaration of Helsinki (as revised in 2013) and complied with ethical standards of national and institutional committees on human experimentation. Informed consent for use of personal information was obtained in writing for every patient involved in the study through a designated form to be signed at the time of the first visit.

Criteria of exclusion from the analysis were the following: high-order (>2 fetuses) multiple gestations, preexisting diabetes mellitus, pregnancy with miscarriage or therapeutic abortion, referral to our Centre after 14 weeks of gestation, delivery in other hospitals, and limited availability of data. 

Gestational age was derived from the last menstrual period and confirmed by first-trimester ultrasound, during which chorionicity was also established by applying standard ultrasonographic criteria [25,31]. After the diagnosis of twin pregnancy, patients were referred to TPCU and received thorough counseling regarding the peculiarities of twin gestation, the risk of maternal and fetal complications, and the follow-up protocol.

Clinical examinations were scheduled monthly and included: measurement of blood pressure, prescription of lab exams such as blood count, urine test and culture, serology for main prenatal infections, and cervical–vaginal swab (repeated in case of positivity). Since the first visit to TPCU, all patients with twin pregnancies were treated with oral low-dose acetylsalicylic acid (ASA), 100 mg/die, to prevent hypertensive disorders of pregnancy. They were also given dietary recommendations in order to appropriately limit weight gain and guarantee a correct intake of macro and micronutrients [32].

All patients with uncomplicated, dichorionic twin pregnancies underwent serial growth-assessing ultrasound (US) examinations every 4 weeks starting from week 20, while monochorionic twin pregnancies received US examinations every 2 weeks from week 16. During US examination, in monochorionic twin pregnancies, amount of amniotic fluid, and Doppler velocimetry of the umbilical and middle cerebral arteries were also assessed, as recommended [31,33,34]. If any anomaly was observed, US examinations were performed more frequently. At 19–21 weeks, a US scan for detailed anatomy assessment was performed; in addition, monochorionic twin pregnancies also received a fetal echocardiogram.

As per our protocol [35], uncomplicated dichorionic twin pregnancies were offered elective birth between 37 + 0 and 37 + 6 weeks, whereas monochorionic twin pregnancies were scheduled to deliver between 36 + 0 and 36 + 6 weeks. A course of antenatal corticosteroids aimed at preventing neonatal RDS was offered to patients undergoing elective cesarean section. 

Throughout the study period, fasting plasma glucose was measured in all pregnant women in the first trimester in order to identify high-risk women without anamnestic risk factors. GDM screening was performed following the one-step approach consisting of 75 g, 2 h oral glucose tolerance test (OGTT) (cutoff values: basal fasting plasma glucose ≥ 92 mg/dL; at 1 h ≥ 180 mg/dL; at 2 h ≥ 153 mg/dL), as recommended by the International Association of Diabetes and Pregnancy Study Group (IADPSG) [36] and other guidelines [37]. GDM was defined as ≥1 abnormal OGTT value. As national guidelines recommend [38], OGTT was offered at 24 to 28 weeks to all twin pregnancies and to singleton pregnancies with risk factors (BMI > 30 kg/cm^2^, previous baby weighing 4.5 kg or more, previous GDM, family history of diabetes mellitus, ethnicity with a high prevalence of diabetes and/or fasting plasma glucose between 5.6 and 6.9 mmol/L in the first trimester). Women who had GDM in a previous pregnancy were offered early OGTT screening at 16 to 18 weeks, which was then repeated at 24 to 28 weeks if the first was negative [38]. 

Once GDM was diagnosed, all patients were immediately referred to the GDM Care Unit of our hospital, underwent nutritional counseling for dietary modifications, and began capillary blood glucose monitoring. Target glycemic levels were set as follows: fasting plasma glucose < 95 mg/dL, 1 h after meal < 140 mg/dL. Patients who could not achieve glycemic control with dietary intervention received insulin therapy at individualized doses.

The following variables were considered: (a) woman’s general features: age, parity, ethnicity, pre-pregnancy body mass index (BMI), mode of conception (spontaneous or with medically assisted reproduction), chorionicity; (b) maternal outcomes: weight gain during pregnancy, hypertensive disorders, need of insulin therapy, insulin dose (UI), intrauterine fetal death (IUFD), mode of delivery, gestational age at birth and OGTT values; (c) neonatal outcomes: APGAR score <7 at 1 and 5 min, frequency of being SGA (<10° centile), or large for gestational age (LGA) (>90° centile), neonatal hypoglycemia. We compared maternal and perinatal outcomes of twin pregnancies complicated by GDM vs. (a) those of singleton pregnancies with GDM, and (b) those of twin pregnancies not complicated by GDM. 

The studied groups were compared using the two-tailed Student’s *t*-test for parametric continuous variables, and the Mann–Whitney U test for non-parametric variables. Categorical data were compared using the χ^2^ or Fisher’s exact tests. We also performed multivariate logistic regression analysis to calculate adjusted odd ratios (aORs), in order to adjust both for differences in maternal characteristics (e.g., maternal age, parity, pre-pregnancy BMI) and for confounders of perinatal outcome (e.g., gestational age and weight at birth). 

Statistical analysis was performed using SPSS Statistical analysis software by SPSS Statistics Inc. IBM Corp (Released 2021. IBM SPSS Version 28.0.1.1). *p*-values < 0.05 were considered statistically significant.

## 3. Results

The study included 1235 twin pregnancies, among which 227 (18.4%) had GDM. Data of 1060 singleton pregnancies with GDM, attending the GDM Care Unit of our center in the same period, were also collected. Overall, outcome data of 2364 twins and 1054 singleton newborns were obtained.

### 3.1. GDM Twin Pregnancies vs. GDM Singleton Pregnancies

Women with GDM twin pregnancy were significantly older (35 ± 5.5 vs. 34 ± 5.0 years; *p* < 0.001) and thinner (BMI 23 ± 4.7 vs. 25 ± 5.8 kg/m^2^; *p* < 0.001) than women with GDM singleton pregnancies; they were also less frequently nulliparous (44.5% vs. 52.8%; *p* = 0.026), and more often Caucasian (96.0% vs. 91.3%; *p* = 0.014) (Table 1). 

In comparison with women with GDM singleton pregnancy, those with GDM twin pregnancy gained more weight during pregnancy (13 ± 5.7 vs. 10 ± 5.8 kg; *p* < 0.001), had a higher rate of preeclampsia (5.7% vs. 2.7%; aOR 2.0, 95% CI: 1.2–3.8) and more frequently underwent cesarean section (82.8% vs. 39.6%; aOR 7.5, 95% CI: 5.2–10.8) (Table 2). As could be expected, the mean gestational age at delivery was significantly lower for GDM twins than singletons (35 ± 2.1 vs. 38 ± 1.8 weeks; *p* < 0.001) (Table 2). Rather surprisingly, insulin therapy was less frequently used in GDM twin pregnancies compared to singletons (11.0% vs. 22.5%; aOR 0.5, 95% CI: 0.3–0.7), though the required dose of insulin was comparable (Table 2).

During OGTT, women with GDM twin pregnancy had a lower incidence of abnormal basal values (56.3% vs. 84.6%; aOR 0.2, 95% CI: 0.1–0.5), comparable 1 h values, and higher incidence of abnormal 2 h values (47.9% vs. 11.5%; aOR 7.1, 95% CI: 3.2–15.7), although with significantly reduced mean glucose levels (163 ± 9.5 vs. 190 ± 14.8 mg/dL) (Table 3).

Newborns of GDM twin pregnancies were significantly less likely LGA than newborns of GDM singleton pregnancies (3.2% vs. 11.9%; aOR 0.2, 95% CI 0.1–0.4) (Table 4). The incidence of a low APGAR score after adjusting for gestational age at delivery was comparable (adjusted *p* = 0.920 and *p* = 0.824 for 1 min and 5 min APGAR scores, respectively). Neonatal hypoglycemia was significantly more frequent in twins than in singleton newborns after a GDM pregnancy (9.1% vs. 1.6%; aOR 2.5, 95% CI 1.1–5.3) (Table 4).

### 3.2. Twin Pregnancy Complicated by GDM vs. Twin Pregnancy without GDM

Women with GDM twin pregnancy were significantly older (35.0 ± 5.5 vs. 33.0 ± 5.0 years; *p* < 0.001) and had higher mean pre-pregnancy BMI (23.0 ± 4.7 vs. 22.0 ± 4.0 kg/m^2^; *p* < 0.001) than those with twin pregnancy not complicated by GDM (Table 5). All the other considered variables were comparable, including the incidence of preeclampsia, gestational age at delivery, and the rate of cesarean section.

Twin pregnancies complicated by GDM had a higher incidence of LGA newborns (3.2% vs. 1.0%; aOR 5.3, 95% CI 1.7–14.8) and a lower rate of low 1 min APGAR score (11.1% vs. 16.8%; aOR 0.5, 95% CI 0.3–0.9) than twin pregnancies without GDM. No differences in the frequency of SGA newborns and low 5 min APGAR score were noticed (Table 6).

## 4. Discussion

The present study evaluated obstetric and neonatal outcomes of twin pregnancies complicated by GDM by comparing them with two control groups: singleton GDM pregnancies and twin pregnancies without GDM. Overall, our data support the idea that GDM does not significantly increase the rate of adverse outcomes in twin pregnancies. 

The incidence of GDM in our sample of twin pregnancies was 18.4%, higher than the one reported by previous studies (nearly 8%) [6,7,16,21,23,29,30], and also higher than the one averagely found in singletons (5–6%) [1,22,23]. This could be explained by the adoption of the 75 g 2 h OGTT to diagnose GDM; this tolerance test, in fact, has higher sensitivity than the two-step approach used in most other studies (50 g oral glucose challenge test—OGCT—followed, in case of positivity, by 100 g OGTT). Indeed, the prevalence of GDM in singleton pregnancies was previously reported at 14.5% when using the one-step OGTT approach vs. 6% when using the two-step OGCT/OGTT approach [39,40,41]. 

The difference we found in maternal age and parity between GDM twin and GDM singleton pregnancies was already reported in previous studies [4,21,42], as well as the significantly lower pre-pregnancy BMI in twin pregnancy [7]. The last observation could reflect a different physiopathology at the basis of GDM onset: in singleton pregnancies, the occurrence of GDM is more strictly tied to lifestyle and, therefore, more frequent in women with higher BMI [1], whereas twin pregnancies develop GDM because of an increased placental mass with higher levels of placental lactogen [4,5,6,7]. However, when comparing twin pregnancies with GDM vs. those without GDM, the former group resulted significantly older and had higher BMI, in agreement with previous data [4,16,19,20,30]: thus, also in twin pregnancy maternal age and BMI may act as independent risk factors for GDM [1,4,16], though with smaller influence than in singletons. Independently from pre-conception BMI, women with twins gained more weight throughout gestation than women with singletons (13 kg vs. 10 kg): this is consistent with the fact that women with GDM twin pregnancy are recommended a slightly more caloric diet than women with a GDM singleton pregnancy, in order to guarantee a sufficient weight gain and decrease the risk of SGA newborns [32,43]. 

As previously reported [13,23], we found an increased risk of preeclampsia in GDM twin pregnancies compared to singletons (aOR 2.0, 95% CI: 1.2–3.8); however, the incidence of preeclampsia was comparable in twin pregnancies with or without GDM. This finding agrees with some previous studies [14,16,19,20], but not with others [13,15,22,44]. It should be noted that all women with twin pregnancies in our study were administered low-dose ASA starting from the first trimester in order to prevent hypertensive disorders of pregnancy: this treatment likely affected the observation. In addition, the higher rate of hypertensive disorders in twin pregnancy compared to singletons is most likely due to twinning per se, and not to GDM; indeed, the relative risk of preeclampsia in twin pregnancy compared to singletons is 2.6 [13,26,45]. 

Twin pregnancies with GDM more frequently ended with cesarean section than singleton counterparts (aOR 7.5, 95% CI: 5.2–10.8), having also significantly lower gestational age at delivery. Again, such differences were not found when comparing twin pregnancies with GDM vs. those without GDM, suggesting that the higher rate of cesarean section and the lower gestational age at birth were related to twinning itself. Published data on this subject are inconsistent [19,20,22,29,30], but in most studies, GDM did not show any association with preterm birth [14,22,29]. 

As concerns neonatal outcomes, differently from other studies [21,23], we observed no difference in APGAR score between twins and singletons with GDM after adjusting data for gestational age at birth, which is the main cause of a low APGAR score. In our sample, GDM twins showed a reduced risk of low APGAR scores at 1 min compared to counterparts without GDM (aOR 0.5, 95% CI 0.3–0.9); also, other studies suggested a protective role of GDM against low 5 min APGAR score for twins [18,20,21,23,30]. These data suggest that not only GDM does not increase the risk of low APGAR score in twin pregnancy, but it could even exert a positive influence, possibly via increased glucose availability for the fetus during the second stage of labor.

Twins were less likely to be LGA (aOR 0.2, 95% CI 0.1–0.4) than singletons, as previously reported [21], but twin pregnancies complicated by GDM showed a higher rate of LGA newborns compared to those without GDM (aOR 5.3, 95% CI 1.7–14.8), in accordance with previously published studies [30]. Indeed, twin pregnancies usually have asymmetrical and decelerated fetal growth from week 32 onward [46], probably due to adaptation to the limited size of uterine cavity [47]. Moreover, twin pregnancies generally have delivery at a lower gestational age, of which low birth weight is a direct consequence. On the other side, we observed no difference in the rate of SGA between twin pregnancies with or without GDM, as previously reported [14,16,19,29]; indeed, a couple of previous studies even reported a protective role of GDM against SGA [18,30]. Taken together, these findings suggest the existence of a higher availability of energy and nutrients in pregnancies with GDM, which could increase the likelihood of being LGA and reduce that of being SGA. However, on this matter, additional data should be gathered, and it should be noted that we do not know how exposure to a hyperglycemic environment in utero could impact metabolism and epigenetics long-term [17].

We found an increased risk of neonatal hypoglycemia for twin pregnancies with GDM compared to singleton counterparts; even Sheehan [17] found a strong association of neonatal hypoglycemia to GDM, and in his study preterm birth and low birth weight, both extremely common in twins, were the strongest predictors of neonatal hypoglycemia.

By comparing OGTT in twin pregnancies with GDM vs. those in singleton counterparts, we found that twin pregnancy had a lower rate of abnormal basal OGTT values and a higher rate of abnormal 2 h OGTT values, with no difference at 1 h; this finding suggests that high levels of plasma glucose are maintained longer in twin pregnancy. When looking at differences in OGTT plasma glucose levels between twin and singleton pregnancy, differently from other studies [5,7,24] we only found reduced levels at 2 h in twins: this may be explained by the fact that we offered OGTT with 75 g to all twin pregnancies, whereas in previous studies only patients positive to the 50 g OGCT underwent OGTT with 100 g. Similarly to what was previously published, in our series insulin treatment was required less frequently in GDM twin pregnancies than in singletons, and the required dose was similar [4,7,24].

Overall, the main strengths of our study are: (a) that all patients were managed by the same team of doctors, who homogeneously applied a specific protocol, and (b) that we could use two different control groups with whom better compare outcome data of GDM twin pregnancies. Limitations of the study include, besides its retrospective nature, the complete lack of information about glycemic control, a factor that can deeply affect pregnancy outcomes. Additionally, the fact that OGTT was offered to women with singleton pregnancies only if they had risk factors could create a bias. However, since multiple pregnancies are at higher risk for GDM per se, offering OGTT to women with singleton pregnancy and no other risk factor would create the opposite bias: they would be at a much lower risk of hyperglycemia and consequent complications. It should be noted that fasting plasma glucose was measured in all singleton pregnancies (also those without risk factors), to detect early alterations in glucose metabolism. In addition, multivariate logistic regression analysis was performed in order to adjust for differences in maternal characteristics (not only pre-pregnancy BMI but also maternal age and parity). Thus, the effects of such bias, if not completely erased, are surely minimized.

In conclusion, our data suggest that GDM does not significantly worsen obstetric and neonatal outcomes of twin pregnancy. Strangely enough, it could also improve some neonatal outcomes that are suboptimal in twin pregnancies, such as birth weight. Our data support the concept that there are significant differences in glucose metabolism between twin and singleton pregnancies; they should be carefully studied and taken into consideration when assessing optimal plasma glucose levels in GDM twin pregnancy. GDM in twin pregnancy should be further explored by larger population studies, with the specific aim of establishing targeted diagnostic criteria, screening times, and specific management protocols.

## Figures and Tables

**Table 1:** Baseline characteristics of women with twin pregnancy complicated by gestational diabete mellitus (GDM twin pregnancy) or singleton pregnancy complicated by GDM (GDM singleton pregnancy). Data are shown as mean ± SD or as number and percentage.

	GDM Twin Pregnancy (n = 227)	GDM Singleton Pregnancy (n = 1060)	*p*-Value
Age	35.0 ± 5.5	34.0 ± 5.0	<0.001
Nulliparity	101 (44.5%)	560 (52.8%)	0.026
BMI * (kg/m^2^)	23.0 ± 4.7	25.0 ± 5.8	<0.001
Caucasian ethnicity	218 (96.0%)	968 (91.3%)	0.014

* BMI = body mass index.

**Table 2:** Comparison between outcomes of GDM twin pregnancies vs. GDM singleton pregnancies. Data are shown as mean ± SD or as number and percentage. aOR = adjusted odds ratio.

	GDM Twin Pregnancy (n = 227)	GDM Singleton Pregnancy (n = 1060)	*p*-Value	aOR (95% CI)
Preeclampsia	13 (5.7%)	29 (2.7%)	0.035	2.0 (1.2–3.8)
Insulin therapy	25 (11.0%)	239 (22.5%)	<0.001	0.5 (0.3–0.7)
Insulin daily dose (IU)	12 ± 10	12 ± 9	0.853	-
Weight gain (kg)	13 ± 5.7	10 ± 5.8	<0.001	-
Gestational age at delivery (weeks)	35 ± 2.1	38 ± 1.8	<0.001	-
Cesarean section	188 (82.8%)	420 (39.6%)	<0.001	7.5 (5.2–10.8)

**Table 3:** Comparison of 75 g OGTT values in GDM twin pregnancies vs. GDM singleton pregnancies.

	GDM Twin Pregnancy (n = 227)	GDM Singleton Pregnancy (n = 1060)	*p*-Value	aOR (95% CI)
Abnormal basal OGTT * values	54 (56.3%)	66 (84.6%)	<0.001	0.2 (0.1–0.5)
Basal plasma glucose level	5.5 ± 0.32 (mmol/L) 99.8 ± 5.8 (mg/dL)	5.7 ± 0.49 (mmol/L) 102.0 ± 8.8 (mg/dL)	0.709	-
Abnormal 1 h OGTT value	30 (31.3%)	20 (25.6%)	0.416	1.3 (0.7–2.6)
1 h plasma glucose level	10.9 ± 0.88 (mmol/L) 197 ± 16.0 (mg/dL)	11.3 ± 1.29 (mmol/L) 204 ± 23.3 (mg/dL)	0.656	-
Abnormal 2 h OGTT value	46 (47.9%)	9 (11.5%)	<0.001	7.1 (3.2–15.7)
2 h plasma glucose level	9.0 ± 0.52 (mmol/L) 163 ± 9.5 (mg/dL)	10.5 ± 0.82 (mmol/L) 190 ± 14.8 (mg/dL)	0.044	-

* OGTT = Oral glucose tolerance test.

**Table 4:** Comparison of neonatal outcome between twin and singleton newborns after pregnancies complicated by GDM. Data are shown as mean ± SD or as number and percentage.

	GDM Twin Pregnancy Newborns (n = 440)	GDM Singleton Pregnancy Newborns (n = 1054)	*p*-Value	aOR (95% CI)
LGA *	14 (3.2%)	126 (11.9%)	<0.001	0.2 (0.1–0.4)
APGAR 1’ < 7	49 (11.1%)	46 (4.3%)	0.920	1.2 (0.6–1.5)
APGAR 5’ < 7	11 (2.5%)	9 (0.9%)	0.824	1.1 (0.5–2.6)
Neonatal hypoglycemia	40 (9.1%)	17 (1.6%)	0.023	2.5 (1.1–5.3)

* LGA = large for gestational age (>90° centile).

**Table 5:** Baseline characteristics of women with twin pregnancy complicated by GDM (GDM twin pregnancy) or twin pregnancy not complicated by GDM (Twin pregnancy—No GDM). Data are shown as mean ± SD or as number and percentage.

	GDM Twin Pregnancy (n = 227)	Twin Pregnancy—No GDM (n = 1008)	*p*-Value	aOR (95% CI)
Age	35.0 ± 5.5	33.0 ± 5.5	<0.001	-
Spontaneous conception	158 (69.6%)	755 (74.9%)	0.101	-
Dichorionic twinning	135 (59.5%)	597 (59.2%)	0.946	-
Nulliparity	101 (44.5%)	390 (38.6%)	0.087	-
BMI (kg/m^2^)	23.0 ± 4.7	22.0 ± 4.0	<0.001	-
Caucasian ethnicity	218 (96.0%)	927 (92.0%)	0.098	-
Preeclampsia	13 (5.7%)	62 (6.2%)	0.183	0.6 (0.3–1.3)
IUFD *	2 (0.9%)	12 (1.2%)	0.268	0.8 (0.6–1.2)
Gestational age at delivery (weeks)	35.0 ± 2.1	35.0 ± 2.5	0.377	-
Cesarean section	188 (82.8%)	776 (77.0%)	0.104	1.2 (0.9–1.8)

* IUFD = intrauterine fetal death.

**Table 6:** Comparison of the neonatal outcome of twin newborns of pregnancies complicated by GDM or without GDM. Data are shown as mean ± SD or as number and percentage.

	GDM Twin Pregnancy Newborns (n = 440)	Twin Pregnancy—No GDM Newborns (n = 1924)	*p*-Value	aOR (95% CI)
SGA *	82 (18.6%)	376 (19.5%)	0.833	0.9 (0.7–1.2)
LGA	14 (3.2%)	20 (1.0%)	0.016	5.3 (1.7–14.8)
APGAR 1’ < 7	49 (11.1%)	324 (16.8%)	0.005	0.5 (0.3–0.9)
APGAR 5’ < 7	11 (2.5%)	62 (3.2%)	0.117	0.5 (0.2–1.6)

* SGA = small for gestational age (<10° centile).

## Data Availability

The data presented in this study are available on request from the corresponding author. The data are not publicly available due to privacy reasons.
